# Supplementary material for: Fire, environmental and anthropogenic controls on pantropical tree cover
Source: Commun Earth Environ. 2024 Nov 18;5(1):714. doi: 10.1038/s43247-024-01869-8 (PMC11573711; doi:10.1038/s43247-024-01869-8)
Supplement: Supplementary file 2 — Supplementary Information [file 43247_2024_1869_MOESM2_ESM.pdf]

Supplementary Information: Fire, environmental and anthropogenic controls on pantropical tree cover

Douglas I. Kelley[1*], France Gerard[1], Ning Dong[2,3*], Chantelle Burton[4], Arthur Argles[4], Guangqi Li[5,6], Rhys Whitley[7], Toby R. Marthews[1], Eddy Roberston[4], Graham P. Weedon[8], Gitta Lasslop[9], Richard J. Ellis[1], Ioannis Bistinas[10], Elmar Veenendaal[11]

[1]UK Centre for Ecology and Hydrology, Wallingford OX10 8BB, U.K.

[2]College of Resources and Environment, Huazhong Agricultural University, Wuhan 430070 , China

[3]Georgina Mace Centre for the Living Planet, Imperial College London, Department of Life Sciences, Silwood Park Campus, Ascot SL5 7PY, U.K.

[4]Met Office Hadley Centre for Climate Science and Services, Exeter, U.K.

[5]Department of Geography and Environmental Science, University of Reading, Reading, U.K.

[6]Biological and Environmental Sciences, University of Stirling, Stirling, U.K.

[7]Natural Perils Pricing, Consumer Insurance, Suncorp, Australia

[8]Met Office, Joint Centre for Hydro-Meteorological Research (JCHMR), Crowmarsh Gifford, Wallingford, Oxfordshire OX10 8BB, U.K.

[9]Senckenberg Biodiversity and Climate Research Centre, Frankfurt, Germany

[10]Cognizant Benelux BV, Paul van Vlissingenstraat 10, 1096BK, Amsterdam, The Netherlands

[11]Plant Ecology and Nature Conservation Group, Wageningen University, Wageningen, The Netherlands

*** Correspondence:**
Douglas I Kelley
doukel@ceh.ac.uk

Ning Dong
n.dong@mail.hzau.edu.cn

Supplementary Notes 1 ***Benchmarking***

$P(X|\beta)$ provides a useful performance measure, and we used it to assess the relative performance of different burnt area and precipitation datasets and rainfall distribution metrics. However, this, by definition, provides more weight to differences between observation and simulation at extreme tree cover values. To assess non-parametric biases in tree cover and for consistency with the vegetation modelling community, we use the Normalised Mean Error (*NME*) metric [1]. This is slightly different to the Manhattan Metric (*MM*) used to benchmark vegetation cover by the fire modelling community [2,3] as Forrest et al. [4] demonstrated that, for single type cover comparisons (i.e., comparison tree cover), using *NME* is proportional to MM but a more intuitive metric score. *NME* measures the absolute distance between observations (*obs*) and the tree cover reconstructed from a parameter set ($sim(\beta)$) weighted by cell area (A_i) and normalised by variation about the aire weigthed mean of *obs* ($\overline{obs}$).

The NME calculation occurs in three steps:

NME Step 1: Initial comparison between model and observations.

The first step computes the NME based on the absolute area-weighted difference between observed and modeled tree cover values:

$$NME_1(\{obs_i\},\{sim(\beta)_i\}) = \frac{\sum_i A_i \times |\, sim(\beta)_i - obs_i)|}{\sum_i A_i \times |\, \overline{obs} - obs_i)|} \qquad (13)$$

NME Step 2: Removal of mean bias.

The second step eliminates the mean bias from both observations and model simulations by subtracting the mean from the data:

$$NME_2(\{obs_i\}, \{mim(\beta)_i\}) = NME_1\left(\{obs_i - \overline{obs}\}, \{mod_i - \overline{sim(\beta)_i}\}\right) \quad (14)$$

Where $\overline{sim(\beta)_i}$ is the area-weighted mean of the simulated values . This step adjusts for systematic biases, allowing a comparison based solely on deviations from the mean.

NME Step 3: Normalization by variance.

The final step normalizes both observations and model values by their absolute variance:

$$NME_3(\{obs_i\}, \{sim(\beta)_i\}) = NME_1\left(\frac{\{obs_i - \overline{obs}\}}{V(\{obs_i\}}), \frac{\{mod_i - \overline{sim(\beta)_i}\}}{V(\{sim(\beta)_i\})}\right) \quad (15)$$

Where $V(\{x_i\}) \quad = \frac{\sum_i A_i \times |\bar{x} - x_i|}{\sum_i A_i}$

This step standardizes the data, enabling a more precise comparison of variability between the modeled and observed tree cover.

For all steps, a perfect match to observations is 0, with increasing *NME* scores representing decreasing performance. We use three null models to help interpret the score (Supplementary Fig. 2). The mean null model is the score obtained by comparing the mean of all observations with the observations [1]. The best "single value" model is obtained by comparing the median of the observations to observations [5], and its score is, by definition, less than or equal to the mean model score. We also compare randomly resampled observations (without replacement) to the observations [1]. As this is different depending on the resampling order, we perform 1000 bootstraps to describe the distribution of our randomly resampled null model. As our posterior is also represented by a distribution of tree covers for each cell, we can, therefore, describe the amount by which our posterior beats each of our null models (Supplementary Fig. 2) or the fraction overlap with randomly resampled data.

When performing comparisons in Supplementary Fig. 2, the parameters β, used in $sim(\beta)$, are drawn from the model's posterior parameter distribution.

Supplementary Notes 2 ***Framework evaluation***

Reconstructed tree cover reproduces the magnitude and spatial pattern of VCF tree cover (Supplementary Fig. 4), with relatively little spread associated with parameter uncertainty (Supplementary Fig. 2, Supplementary Fig. 3). The framework tends to overestimate tree cover in savanna areas and slightly underestimate cover in arid and forest areas (Table 1, Supplementary Fig. 4). This follows the same pattern found where VCF underestimates tree cover in savanna and overestimates in forest/arid regions. However, tree cover mismatch between the framework and VCF in savannas are small and non-significant - falling between 20-80% of the framework full posterior (Supplementary Fig. 4). This is reflected in the framework's benchmarking performance, with an NME score of 0.28-0.32 - a 68-72% improvement on the best null model. The framework also reproduced the bimodal distribution found in VCF (Supplementary Fig. 2), though with the second peak slightly shifted, occurring at 98% tree cover instead of 95%.

Runs with MDDM and MADD rainfall distribution metrics perform slightly better (0.27-0.32 and 0.28-0.31) than with other metrics, though there is no significant difference in their metric scores. This slightly better performance can also be seen in MDDMs narrower model error (Supplementary Fig. 3), making up 26% of the full framework posterior. There are small but significant differences in performances from runs with different precipitation datasets - with MSWEP combined with either GFED4 or GFED4s (0.27-0.28) performing best, with a 15-16%

improvement on the worse combination (CMORPH with MCD45). MSWEP has also performed better than the other rainfall products in hydrological comparisons [6,7].

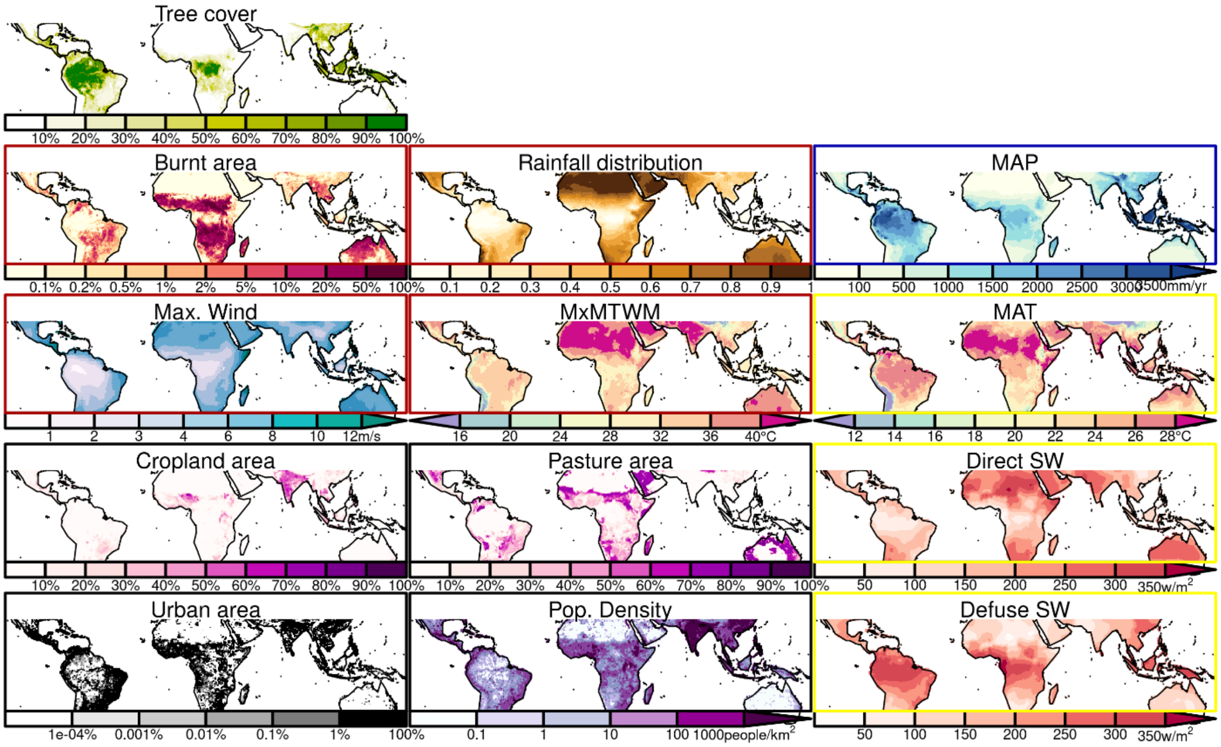

Supplementary Figure 1. Variables used in optimisation. (top) The framework reconstructed tree cover from: “Stresses” (red boxes, top left) driven by burnt area from GFED4s [8], but also using the different datasets on (Supplementary Fig. 6) in alternate optimisations; rainfall distribution using the MADD metric with MSWEP precipitation [9], but also using the different metric and data combinations (Supplementary Fig. 8), 90% maximum monthly wind from CRU-NCEP [10], and mean maximum temperature of the warmest month (MxMTWM) from CRUTS4.03 [11]; human pressures (black box) cropland, pasture, and urban area from HYDEv3.1 [12]. Mean Annual Precipitation (MAP) (blue box) here from MSWEP [9], but also using alternate data (Supplementary Fig. 8); Energy (yellow box) combining Mean Annual Temperature (MAT) from CRUTS4.03 [11], and direct and defuse SW derived from CRUTS4.03 cloud cover [11] using SLASH [13].

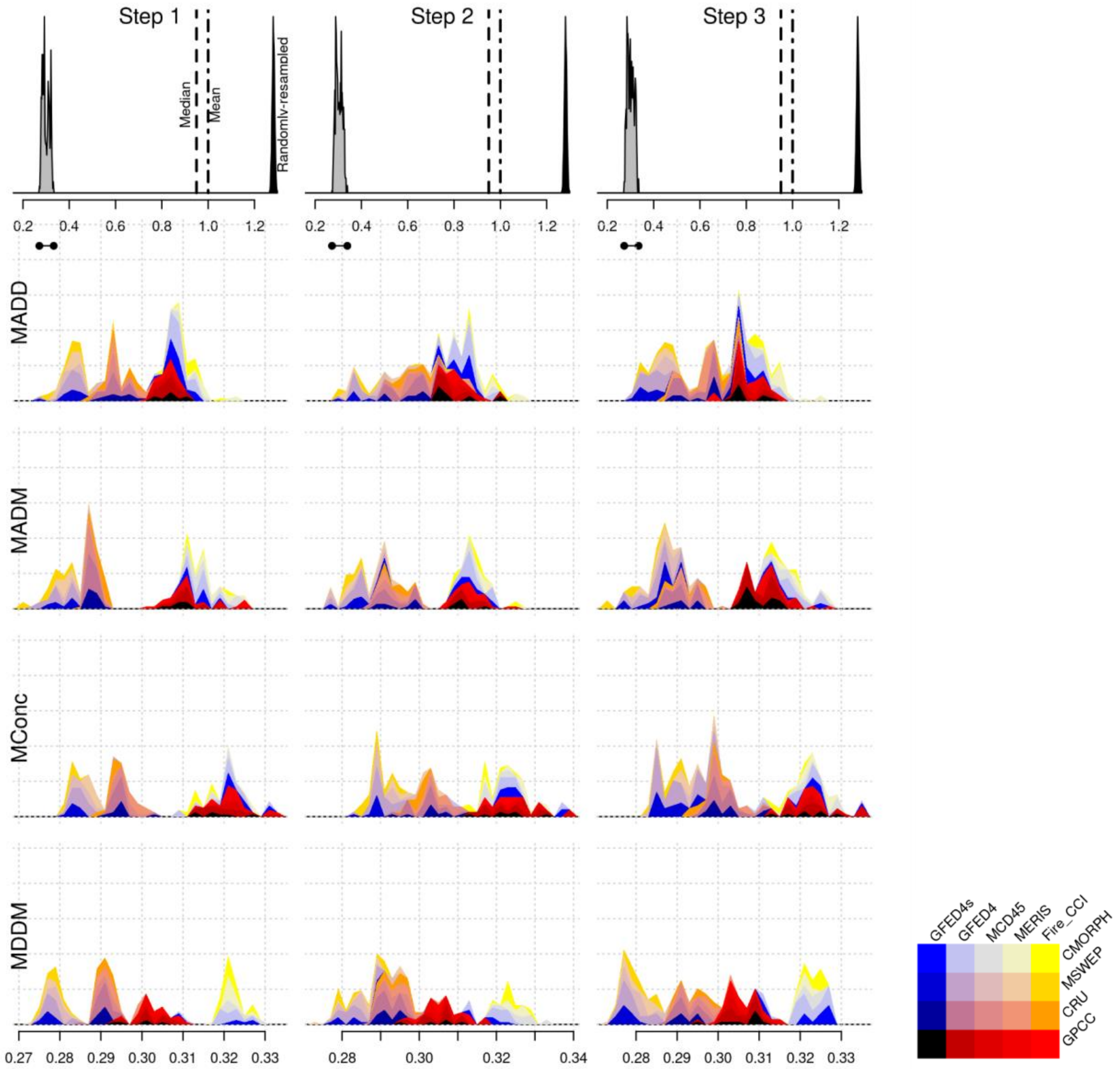

Supplementary Figure 2. NME step1-3 metrics scores. Top: the frameworks posterior (histogram, generated from 25,000 samples) and median (dashed line), mean (dot-dashed line), and randomly resampled (black) null models. Dumbell on the x-axis shows the x-axis range on subsequent rows, which display scores for individual rainfall distribution metrics coloured by rainfall and burnt area datasets.

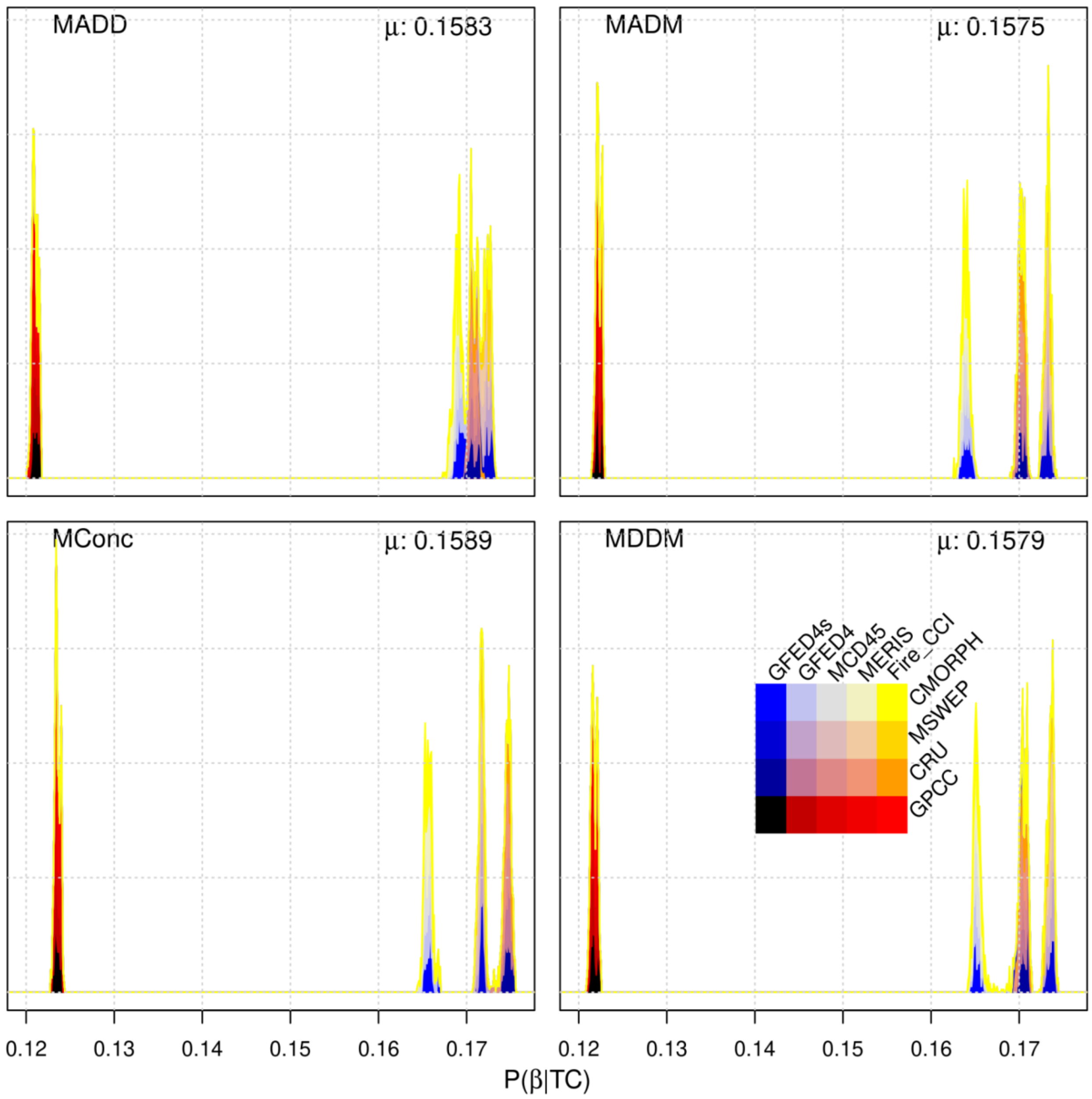

Supplementary Figure 3. Probability distribution of the framework (i.e. $P(\beta|X)$ for (panels) rainfall distribution metric and (colours) burnt area and precipitation datasets.

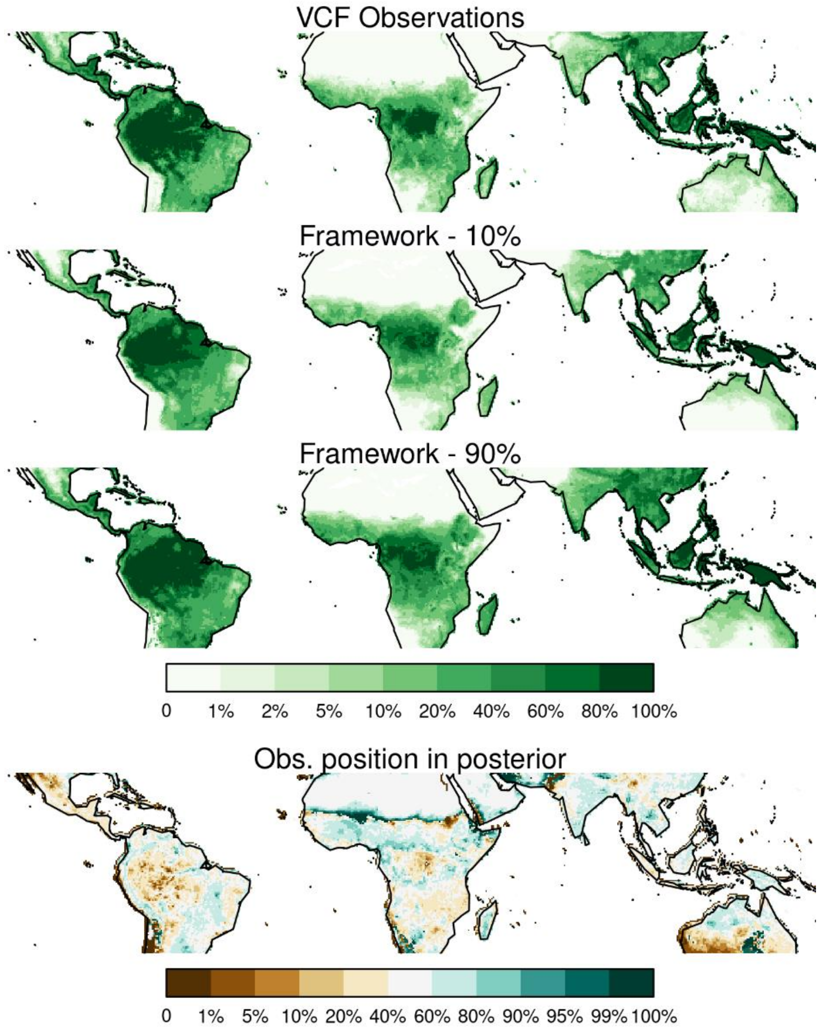

Supplementary Figure 4. Percentage tree cover in framework vs. observations. From (top-bottom) VCF observations[14], 10% and 90% percentiles of the framework, accounting for model parameter uncertainty and (bottom map) positions of the observations in the framework's full posterior. Brown indicates underestimation, and blue indicates overestimation.

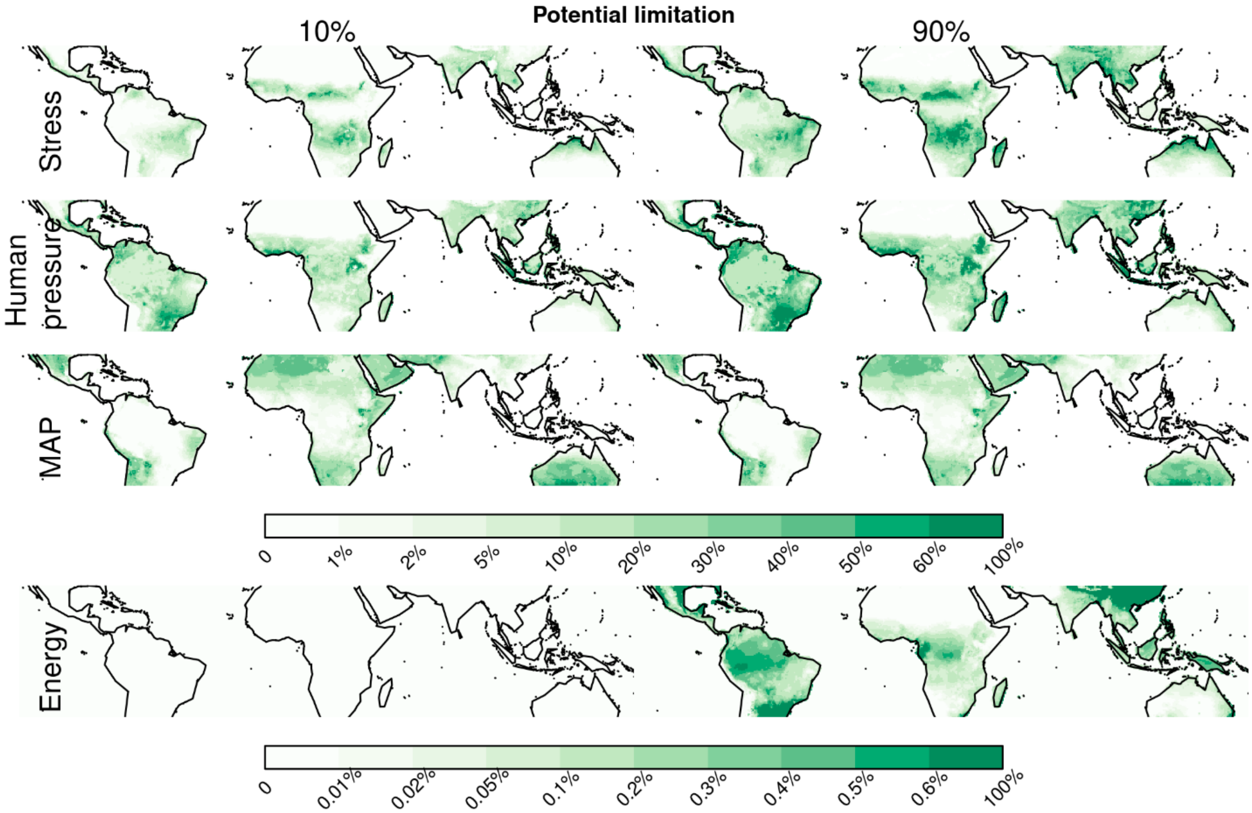

Supplementary Figure 5. Potential increase in percentage tree cover if each limiting factor were remove

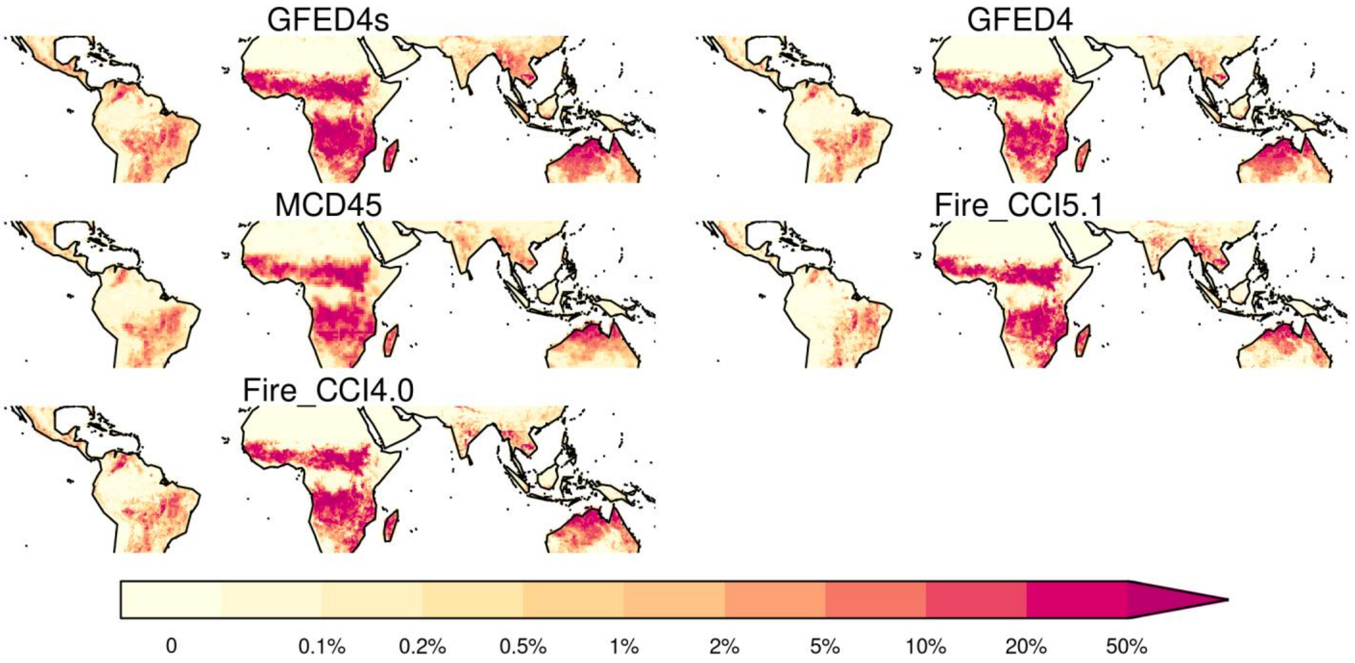

Supplementary Figure 6. The five annual burnt area products used in this study from the Fire Model Intercomparison Project[3]**.** See Supplementary Table 2 for dataset reference.

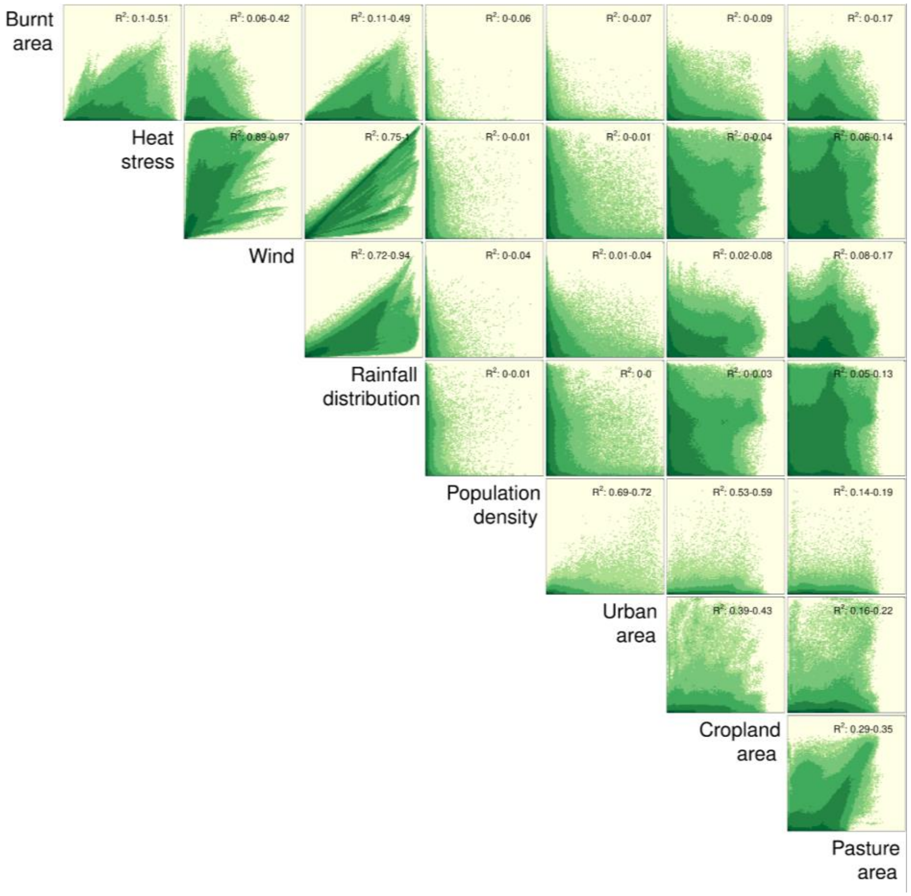

Supplementary Figure 7. Co-variation of the impact of environmental stresses and human pressures on tree cover. Axis are the relative impact on tree cover by the labelled controls d variable, and the intensity of the plot shows the density of points.

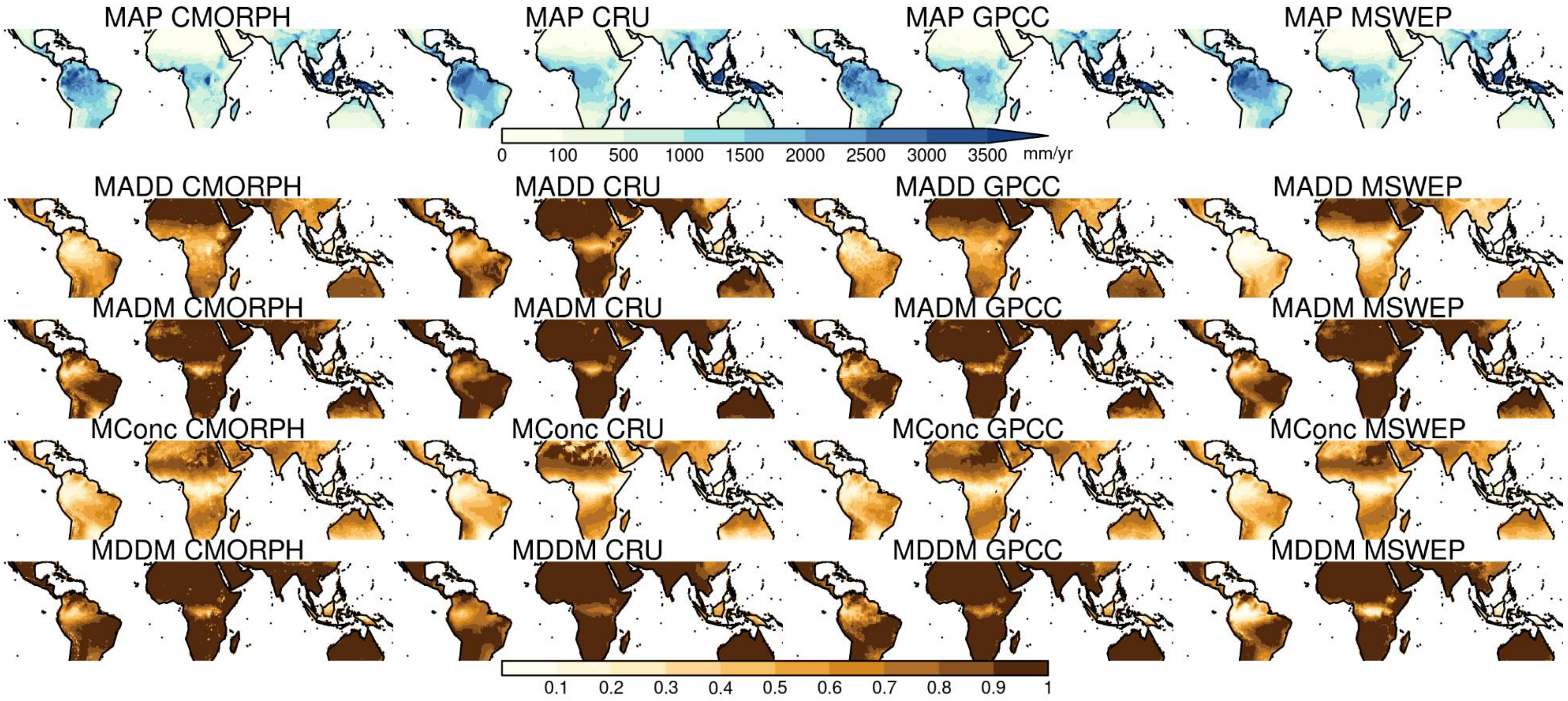

Supplementary Figure 8. Precipitation and rainfall distribution drivers. Top row four different Mean Annual Precipitation (MAP) products used in separate optimisations with corresponding rainfall distribution metrics mapped below. See Supplementary Table 2 for dataset reference and "Datasets" in methods for rainfall distribution definitions.

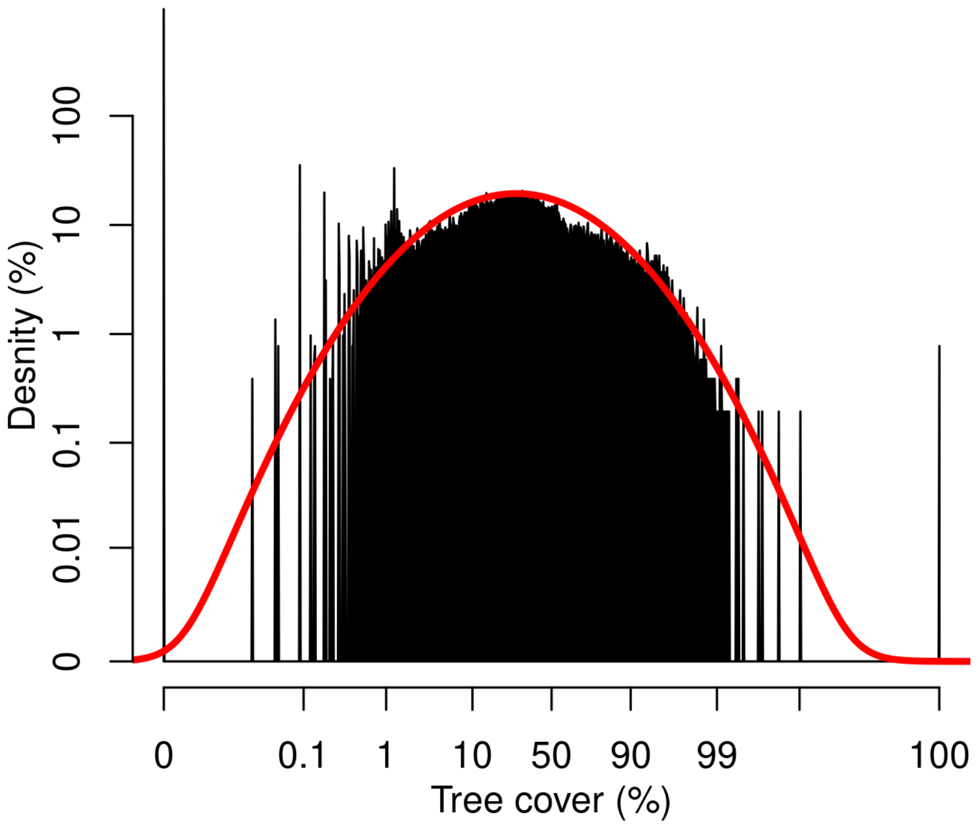

Supplementary Figure 9. Distribution of VCF tree cover under a logit transformation. The Red line shows a normal distribution best fit against tree cover greater than 0.

Supplementary Table 1: Probability of tropics-wide impact of variable in the column exceeding the impact of variable in the row.

	MAP	Stress	Human pressures	Burnt area	Heat stress	Wind	Rainfall distribution	Population density	Urban area	Cropland area	Pasture area
All tropics											
**MAP**		0.17	0.62	0	0.16	0.001	0.16	0	0	0.025	0.14
**Stress**	0.83		0.97	0	0.002	0	0.002	0.001	0.002	0.007	0.32
**Human pressures**	0.38	0.031		0	0.005	0	0.003	0	0	0	0
**Burnt area**	1	1	1		0.99	0.94	0.99	0.1	0.6	0.99	1
**Heat stress**	0.84	1	1	0.01		0.076	0.44	0.003	0.015	0.17	0.64
**Wind**	1	1	1	0.061	0.92		0.99	0.001	0.016	0.94	1
**Rainfall dist.**	0.84	1	1	0.01	0.57	0.008		0.003	0.006	0.013	0.59
**Pop. density**	1	1	1	0.9	1	1	1		1	1	1
**Urban area**	1	1	1	0.4	0.99	0.98	0.99	0		1	1
**Cropland area**	0.98	0.99	1	0.006	0.83	0.058	0.99	0	0		1
**Pasture area**	0.86	0.68	1	0.002	0.36	0	0.41	0	0	0	
Savanna & grassland											
**MAP**		0.69	0.9	0.021	0.37	0.015	0.46	0	0	0.002	0.42
**Stress**	0.31		0.71	0	0.002	0	0.002	0.001	0.001	0.004	0.11
**Human pressures**	0.1	0.29		0	0.12	0	0.16	0	0	0	0
**Burnt area**	0.98	1	1		0.98	0.77	0.99	0.003	0.055	0.88	0.99
**Heat stress**	0.63	1	0.88	0.02		0.072	0.49	0	0.005	0.067	0.46
**Wind**	0.99	1	1	0.23	0.93		0.99	0.001	0.001	0.49	1
**Rainfall dist.**	0.54	1	0.84	0.013	0.51	0.008		0.002	0.003	0.011	0.3
**Pop. density**	1	1	1	1	1	1	1		1	1	1
**Urban area**	1	1	1	0.95	1	1	1	0		1	1
**Cropland area**	1	1	1	0.12	0.93	0.51	0.99	0	0		1
**Pasture area**	0.58	0.89	1	0.007	0.54	0	0.7	0	0	0	

Supplementary S2. Limiting factors there contributing controls and data sources.

Limiting factor	Control	Variable	Source	Reference
Stress	Fire	Burnt area	GFED4s	[8]
			GFED4	[15]
			MCD45	[16]
			Fire_CCI4.0	[17]
			Fire_CCI5.1	[18]
	Heat stress	Mean max. temperature of the warmest month	CRUTS4.03	[11]
	Windthrow	90% monthly windspeed	CRU-NCEP	[10]
	Rainfall distribution	Mean annual dry days (MADD)	Same as MAP	
		The mean number of Dry days in the Driest Month (MDDM)		
		Mean Annual Precipitation in the Driest Month (MADM)		
		Mean Concentration (MConc)		[19]
Human pressures	Urban area		HYDE	[12]
	Cropland			
	Pasture			
	Population density			
MAP*	MAP	Mean Annual Precipitation	CRUTS4.03	[11]
			MSWEP	[9]
			CMORPH	[20,21]
			GPCC	[22]
MAT	MAT	Mean Annual Temperature	CRUTS4.03	[11]
Shortwave	Direct	Incoming radiation partitioned by the SPLASH model		[13]
	Diffuse			

Supplementary References

1. Kelley, D. I., Harrison, S. P., Wang, H. & Simard, M. A comprehensive benchmarking system for evaluating global vegetation models. (2013).

2. Lasslop, G. *et al.* Global ecosystems and fire: Multi-model assessment of fire-induced tree-cover and carbon storage reduction. *Glob. Chang. Biol.* **26**, 5027–5041 (2020).

3. Hantson, S. *et al.* Quantitative assessment of fire and vegetation properties in simulations with fire-enabled vegetation models from the Fire Model Intercomparison Project. *Geoscientific Model Development* vol. 13 3299–3318 https://doi.org/10.5194/gmd-13-3299-2020 (2020).

4. Forrest, M., Tost, H., Lelieveld, J. & Hickler, T. Including vegetation dynamics in an atmospheric chemistry-enabled general circulation model: linking LPJ-GUESS (v4. 0) with the EMAC modelling system (v2. 53). *Geoscientific Model Development* **13**, 1285–1309 (2020).

5. Burton, C. *et al.* Representation of fire, land-use change and vegetation dynamics in the Joint UK Land Environment Simulator vn4. 9 (JULES). *Geoscientific Model Development* **12**, 179–193 (2019).

6. Beck, H. E. *et al.* Global-scale evaluation of 22 precipitation datasets using gauge observations and hydrological modeling. *Hydrol. Earth Syst. Sci.* **21**, 6201–6217 (2017).

7. Beck, H. E. *et al.* Daily evaluation of 26 precipitation datasets using Stage-IV gauge-radar data for the CONUS. *Hydrol. Earth Syst. Sci.* **23**, 207–224 (2019).

8. van der Werf, G. R. *et al.* Global fire emissions estimates during 1997–2016. *Earth Syst. Sci. Data* **9**, 697–720 (2017).

9. Beck, H. E., Van Dijk, A. & Levizzani, V. MSWEP: 3-hourly 0.25 global gridded precipitation (1979-2015) by merging gauge, satellite, and reanalysis data. *Hydrol. Earth Syst. Sci.* (2017).

10. Harris, I. C. CRU JRA v2. 0: A Forcings Dataset of Gridded Land Surface Blend of Climatic Research Unit (CRU) and Japanese Reanalysis (JRA) Data. (2019).

11. Harris, I., Jones, P. D., Osborn, T. J. & Lister, D. H. Updated high-resolution grids of monthly climatic observations - the CRU TS3.10 Dataset. *Int. J. Climatol.* **34**, 623–642 (2013).

12. Klein Goldewijk, K., Goldewijk, K. K., Beusen, A., Van Drecht, G. & De Vos, M. The HYDE 3.1 spatially explicit database of human-induced global land-use change over the past 12,000 years. *Glob. Ecol. Biogeogr.* **20**, 73–86 (2010).

13. Davis, T. W. *et al.* Simple process-led algorithms for simulating habitats (SPLASH v.1.0): robust indices of radiation, evapotranspiration and plant-available moisture. *Geoscientific Model Development* **10**, 689–708 (2017).

14. Dimiceli, C. *et al.* MOD44B MODIS/Terra Vegetation Continuous Fields Yearly L3 Global 250m SIN Grid V006. NASA EOSDIS Land Processes DAAC, USGS Earth Resources Observation and Science (EROS) Center https://doi.org/10.5067/MODIS/MOD44B.006 (2015).

15. Giglio, L., Randerson, J. T. & van der Werf, G. R. Analysis of daily, monthly, and annual burned area using the fourth-generation global fire emissions database (GFED4). *J. Geophys. Res. Biogeosci.* **118**, 317–328 (2013).

16. Roy, D. P., Boschetti, L., Justice, C. O. & Ju, J. The collection 5 MODIS burned area product — Global evaluation by comparison with the MODIS active fire product. *Remote Sens. Environ.* **112**, 3690–3707 (2008).

17. Alonso-Canas, I. & Chuvieco, E. Global burned area mapping from ENVISAT-MERIS and MODIS active fire data. *Remote Sens. Environ.* **163**, 140–152 (2015).

18. Chuvieco, E. *et al.* Generation and analysis of a new global burned area product based on MODIS 250 m reflectance bands and thermal anomalies. *Earth Syst. Sci. Data* **10**, 2015–2031 (2018).

19. Kelley, D. I. *et al.* A comprehensive benchmarking system for evaluating global vegetation models. (2013).

20. Joyce, R. J., Janowiak, J. E., Arkin, P. A. & Xie, P. CMORPH: A Method that Produces Global Precipitation Estimates from Passive Microwave and Infrared Data at High Spatial and Temporal Resolution. *J. Hydrometeorol.* **5**, 487–503 (2004).

21. Marthews, T. R., Blyth, E. M., Martínez-de la Torre, A. & Veldkamp, T. I. E. A global-scale evaluation of extreme event uncertainty in the eartH2Observe project. *Hydrol. Earth Syst. Sci.* **24**, 75–92 (2020).

22. Schneider, U., Becker, A., Finger, P., Meyer-Christoffer, A. & Ziese, M. GPCC Full Data Monthly Product Version 2018 at 0.5◦: Monthly Land-Surface Precipitation from Rain-Gauges Built on GTS-Based and Historical Data. *Deutscher Wetterdienst: Offenbach am Main, Germany* (2018).
